# Adaptation and Validation of the Bern Illegitimate Tasks Scale (BITS) in the Context of a Portuguese Public University

**DOI:** 10.3390/bs16060954

**Published:** 2026-06-10

**Authors:** Joana Vieira dos Santos, Mariana Marques, Cátia Sousa, Alexandra Gomes, Luis Felipe Lopes

**Affiliations:** 1 Research Centre for Tourism, Sustainability and Well-Being, University of Algarve, 8005-139 Faro, Portugal; 2 University Research Centre in Psychology, University of Algarve, 8005-139 Faro, Portugalcavsousa@ualg.pt (C.S.); asgomes@ualg.pt (A.G.); 3 Department of Administrative Sciences, Center for Social and Human Sciences, Federal University of Santa Maria, Santa Maria 97105-900, Brazil

**Keywords:** illegitimate tasks, psychometric properties, scale adaptation, higher education, Bern Illegitimate Tasks Scale, SOS theory

## Abstract

Illegitimate tasks are assignments that threaten professional identity by not being related to the intrinsic quality or morality of the main profession. This concept has gained attention within the Stress as Offense to Self (SOS) theory, which emphasizes the impact of self-esteem in stressful situations, particularly in the workplace. The SOS theory suggests that self-esteem plays a critical role in how individuals respond to stress: when self-esteem is threatened, it triggers adverse reactions affecting mental, physical, and behavioral dimensions; conversely, strengthening self-esteem promotes well-being. Illegitimate tasks are perceived as unnecessary or unreasonable, varying by profession and non-voluntary in nature, leading to a lack of purpose and meaning for the employee. The Bern Illegitimate Tasks Scale (BITS) was created to assess and quantify these tasks, demonstrating robust psychometric properties across different languages and cultural contexts, including Spanish, Swedish, and Portuguese adaptations. This study aims to translate and adapt the BITS for a public university context characterized by bureaucratic culture. The sample comprises 601 participants from a Portuguese public higher education institution. The translation process followed rigorous procedures to ensure equivalence between the original and Portuguese versions. Data analysis included descriptive statistics, confirmatory factor analysis (CFA), and internal consistency analysis, revealing satisfactory fit indices and high reliability. Despite contextual limitations, the findings affirm the reliability of the adapted scale for application in similar contexts. Future research should aim for more representative samples to enhance generalizability.

## 1. Introduction

Illegitimate tasks are assigned tasks that threaten professional identity by not being related to the intrinsic quality or morality of the main profession (Semmer et al., 2010). This concept has garnered growing attention within the literature on Stress as Offense to Self-theory (SOS; Semmer et al., 2007, 2019).

The SOS theory is a psychological framework that focuses on the impact of self-esteem in stressful situations, particularly in the workplace. Essentially, this theory suggests that self-esteem plays a critical role in how individuals respond to stressful situations encountered in the workplace. When self-esteem is threatened—whether by criticism, failure, or challenging situations—it can trigger a range of adverse reactions that affect not only the mental state but also the physical and behavioral aspects of individuals. Conversely, when self-esteem is strengthened—whether through recognition, achievements, or support—it tends to promote a general state of well-being. Therefore, the SOS theory highlights the importance of recognizing and nurturing employees’ self-esteem in organizational environments. By doing so, organizations can promote a healthier and more productive work climate, in which employees feel valued and capable of effectively coping with the challenges they face (Semmer et al., 2007, 2019).

This theory introduces the concepts of illegitimate tasks and illegitimate stressors—tasks or stressors perceived as unnecessary or unreasonable. Unnecessary tasks are those that could have been avoided, and unreasonable tasks are those outside one’s occupation or not appropriate for that specific profession (Semmer et al., 2010).

Some tasks may be legitimate for one profession but illegitimate for another. What makes them illegitimate is their non-voluntary nature: employees are assigned to these tasks, leading to a lack of purpose and meaning in their work, thus offending one’s self-esteem (Semmer et al., 2010). It is in this context that the Bern Illegitimate Tasks Scale (BITS; Semmer et al., 2010) was created, to systematically assess and quantify the extent to which tasks are perceived as unnecessary or unreasonable. The scale is essential for identifying these stressors, allowing organizations to address and mitigate the harmful effects of illegitimate tasks on employees.

The BITS has already been adapted into several languages and cultural contexts—namely Spanish (Valdivieso Portilla et al., 2021), Swedish (Stengård et al., 2024), and Portuguese (Neves et al., 2023)—demonstrating its broad relevance and applicability. The Spanish version (Valdivieso Portilla et al., 2021) was applied to a sample of 142 nursing staff from a hospital in Guayaquil, Ecuador, demonstrating robust psychometric properties with a Cronbach’s alpha coefficient of 0.857. The Swedish version (Stengård et al., 2024) was applied to 966 human services workers (e.g., teachers and nurses) and 750 non-human service workers (e.g., construction and IT workers), using data from the Swedish Occupational Survey of Health. It revealed acceptable values for McDonald’s Omega: 0.817 for unreasonable tasks and 0.769 for unnecessary tasks. The Portuguese version (Neves et al., 2023) was implemented with a sample of 472 workers across various professional sectors, exhibiting strong psychometric properties (Cronbach’s alpha = 0.923).

These adaptations help ensure that the scale is valid and reliable across different populations. By conducting a new adaptation, we contribute to the broad and effective use of the BITS in specific contexts. In this study, we aim to translate and validate the BITS, applying it to a specific context of a public university. Adapting a scale of illegitimate tasks within a higher education context, characterized by bureaucratic culture, offers several benefits: it enhances the identification of conflicts and promotes transparency by clarifying expectations and roles, thereby improving trust among institutional members. Additionally, this adaptation fosters better communication between faculty, students, and administration.

By addressing illegitimate tasks, institutions can develop policies that create a healthier environment and encourage constructive dialogue about practices and norms. This process aids in conflict management and focuses on improving educational quality, aligning practices with faculty’s needs. Furthermore, clear expectations can reduce stress and promote innovation in pedagogical approaches. Overall, these advantages contribute to a more collaborative and effective teaching environment, even within bureaucratic constraints.

## 2. Materials and Methods

### 2.1. Sample

The sample comprises 601 participants, all workers at a Portuguese public higher education institution. The responses were distributed as follows: Fellow (*n* = 15; 2.5%); Researcher (*n* = 37; 6.2%); Professor (*n* = 436; 72.5%); and Non-Professor Employee (*n* = 113; 18.8%). The respondents were predominantly female (52.2% female; 41.1% male; 2.0% other; 4.7% no response). The most frequent marital status was married or cohabiting (65.2%), followed by single (15.5%), divorced or separated (13.0%), widowed (1.5%), and no response (4.8%). Regarding academic qualifications, most participants held a PhD or third cycle degree (59.3%), followed by a master’s degree or second cycle (21.0%), a bachelor’s degree or first cycle (14.8%), secondary education (4.7%), and primary education (0.2%).

### 2.2. Instrument

The Bern Illegitimate Tasks Scale (BITS; Semmer et al., 2010) measures illegitimate tasks in terms of two dimensions: unreasonable tasks and unnecessary tasks. The scale consists of eight items, rated on a five-point frequency scale: 1 = Never; 2 = Almost never; 3 = Sometimes; 4 = Frequently; 5 = Very frequently. Since this measure had not previously been used with a Portuguese public university sample, it was necessary to adapt and test its metric quality. Accordingly, several quality indicators were examined: face validity, construct validity, and internal consistency.

### 2.3. Translation and Adaptation Procedure

The scale translation process (Appendix A) followed the method proposed by Brislin (1970) to maintain equivalence between the original English measure and the Portuguese version. This procedure comprised four stages: (1) back-translation method; (2) bilingual technique; (3) committee approach; and (4) pre-test procedure. For the pre-test, the translated Portuguese version was administered to a pilot group to assess item clarity and comprehension prior to the main data collection.

### 2.4. Data Collection Procedure

Data were collected through an online self-report questionnaire distributed to all workers of the participating institution. Participation was voluntary and anonymous. Ethical principles regarding informed consent and data confidentiality were observed throughout the study.

### 2.5. Data Analysis

Data analysis was conducted using descriptive statistics, confirmatory factor analysis (CFA), and internal consistency analysis. The CFA was used to evaluate the factor structure of the adapted scale. Model fit was assessed using the following indices: chi-square to degrees of freedom ratio (χ^2^/df), Comparative Fit Index (CFI), Tucker–Lewis Index (TLI), Root Mean Square Error of Approximation (RMSEA), and Standardized Root Mean Square Residual (SRMR). Reliability was assessed using Cronbach’s alpha (α) and McDonald’s omega (ω) coefficients.

## 3. Results

### 3.1. Item Analysis

Table 1 presents the item-level descriptive statistics for the eight BITS items, including means, standard deviations, and item-total correlations. Results indicated that all items presented adequate item-total correlations, and no item required elimination to improve the internal consistency of either subscale.

### 3.2. Confirmatory Factor Analysis

**Figure 1:**
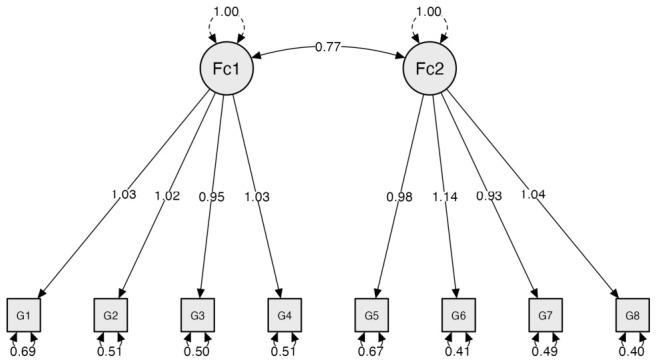
Factorial confirmatory structure of the adapted Bern Illegitimate Tasks Scale (BITS).

Table 2 presents the fit indices for the confirmatory factorial structure tested. The tested model replicates the original two-factor structure of the BITS (see also Figure 1).

The observed χ^2^/df of 9.35 (*p* < 0.001) approached the desired values. The CFI of 0.900 meets the benchmark for good adjustment (Byrne, 2010; Hu & Bentler, 1999). Regarding the SRMR and RMSEA, values lower than 0.05 are considered a good fit, although values close to 0.08 are considered a reasonable adjustment (Schermelleh-Engel et al., 2003). The SRMR value of 0.006 and the RMSEA of 0.17 are discussed further in Section 4.

Figure 1 presents the factorial confirmatory structure of the adapted BITS, displaying the standardized factor loadings for each item and the correlation between the two latent factors (Unnecessary Tasks and Unreasonable Tasks).

### 3.3. Internal Consistency

Scale reliability analysis was performed using Cronbach’s alpha and McDonald’s omega coefficients. The overall reliability across all items was very good (α = 0.916; ω = 0.917), exceeding the values reported in the original article (Study 1: α = 0.83; Study 2: α = 0.88; Semmer et al., 2010).

The following internal consistency values were observed by subscale: Unnecessary Tasks—α = 0.88, ω = 0.88; Unreasonable Tasks—α = 0.89, ω = 0.89. No item required elimination, as doing so would reduce internal consistency in either subscale.

## 4. Discussion

The present study aimed to analyze the factorial structure and psychometric properties of the BITS (Semmer et al., 2010) in a Portuguese public university context (Table A1). Adapting the scale for higher education enhances conflict identification and promotes transparency, improving trust among institutional members and fostering better communication between all stakeholders.

Due to the bureaucratic nature of higher education institutions, there is a prevalent perception among workers that many of their tasks are illegitimate. This perception can lead to frustration and disengagement, as employees feel burdened by responsibilities that do not align with their core roles. The BITS serves as a tool to measure and investigate these perceptions, providing valuable insights into the extent of illegitimate tasks experienced by faculty and staff. By identifying these tasks, it becomes easier to highlight areas for improvement and facilitate discussions about redefining roles and responsibilities within the institution.

Concerning the findings, the reliability of all items was very good, exceeding the values reported in the original article (Semmer et al., 2010). The internal consistency for both subscales—Unnecessary Tasks and Unreasonable Tasks—was also high. The observed fit indices generally approached the desired values, with the CFI indicating good adjustment (Byrne, 2010; Hu & Bentler, 1999).

Despite that, the RMSEA showed a less satisfactory fit. Considering that this index is sensitive to the number of constraints in the model, it is possible that some factor related to the items themselves creates undetected covariance between them (for example, reversed items tend to correlate beyond what would be expected, as they can be somewhat confusing for some participants). This is further supported by the SRMR, which was within acceptable thresholds despite being a similar measure of fit—probably because it is less sensitive to model complexity and the χ^2^ value (Schermelleh-Engel et al., 2003).

Although the values are not optimal, they still indicate a satisfactory fit for the model, similar to the findings of the existing Portuguese adaptation (RMSEA = 0.171; Neves et al., 2023), thereby affirming the reliability of the BITS for application in contexts comparable to those utilised in both studies.

Comparing the present findings to the original study, the results indicate an increase in Cronbach’s alpha values, which is a positive outcome; however, there is also a rise in RMSEA values. Translating a scale involves more than linguistic equivalence—it also requires conceptual and cultural alignment, which can impact the factor structure of the instrument in the new language (Brislin, 1970). The similar RMSEA obtained in a previous Portuguese adaptation (Neves et al., 2023) suggests that contextual factors, such as different interpretations of items or cultural nuances, may be influencing the results.

### Limitations and Future Directions

Although the findings are significant, it is essential to recognize that they are specific to the context in which the study was conducted—a public university characterized by a bureaucratic culture. This contextual limitation restricts the generalizability of the translated and validated scale to other educational settings or non-educational contexts. Therefore, caution is warranted when attempting to extrapolate these findings to different populations or environments, as they may not produce the same outcomes or insights.

Future research should aim to utilize a more representative and diverse sample to enhance the applicability of the results across broader contexts. Additionally, longitudinal studies examining the predictive validity of the adapted BITS in relation to outcomes such as burnout, turnover intention, and organizational commitment would further strengthen the evidence base for its use in Portuguese higher education settings.

## 5. Conclusions

This study successfully adapted and validated the Bern Illegitimate Tasks Scale (BITS) for use in a Portuguese public university context. The adapted scale demonstrated very good overall reliability (α = 0.916; ω = 0.917) and acceptable model fit. Both subscales—Unnecessary Tasks and Unreasonable Tasks—showed high internal consistency. These findings support the use of the BITS as a reliable measure for assessing perceptions of illegitimate tasks in Portuguese higher education institutions.

Identifying and addressing illegitimate tasks in higher education can contribute to healthier organizational environments, reduced stress, improved role clarity, and better communication among faculty, staff, and administration. The adapted BITS provides practitioners and researchers with a validated tool to investigate these phenomena in similar bureaucratic and educational contexts.

## Figures and Tables

**Table 1:** Item descriptive statistics and item-total correlations.

Item	M	SD	Corrected Item-Total r	α If Item Deleted
1. Unnecessary				
Item 1	2.84	1.02	0.67	0.85
Item 2	2.71	0.98	0.70	0.84
Item 3	2.93	1.06	0.73	0.83
Item 4	2.67	1.01	0.68	0.85
2. Unreasonable				
Item 5	2.11	0.94	0.71	0.86
Item 6	1.98	0.91	0.73	0.86
Item 7	2.24	1.03	0.69	0.87
Item 8	2.08	0.97	0.74	0.85

Note: M = mean; SD = standard deviation; r = item-total correlation; α = Cronbach’s alpha.

**Table 2:** Confirmatory factorial structures tested.

Model	χ^2^/df	CFI	TLI	RMSEA	SRMR
1. Bifactorial	9.35	0.900	0.850	0.17	0.006

Note: CFI = Comparative Fit Index; TLI = Tucker–Lewis Index; RMSEA = Root Mean Square Error of Approximation; SRMR = Standardized Root Mean Square Residual.

## Data Availability

The data presented in this study are available on request from the corresponding author. The data are not publicly available due to privacy restrictions.

## Appendix A

Table A1 presents the complete bilingual version of the BITS instrument as adapted for the Portuguese public university context.

**Table A1:** Bern Illegitimate Tasks Scale (BITS)—Original and Portuguese versions.

Item	Original Version (English)	Portuguese Version
	Do you have tasks to take care of, which keep you wondering if…	Tem tarefas a realizar que o deixam a pensar se…
1	…they have to be done at all?	…têm mesmo que ser feitas?
2	…they make sense at all?	…fazem sentido?
3	…they would not exist (or could be done with less effort), if organised differently?	…não existiriam (ou poderiam fazer-se com menos esforço) se estivessem organizadas de maneira diferente?
4	…they just exist because some people simply demand it this way?	…só existem, porque algumas pessoas simplesmente as exigem?
	Do you have work tasks to take care of, which you believe…	Tem tarefas de trabalho a realizar, que acredita que…
5	…should be done by someone else?	…deveriam ser feitas por outra pessoa?
6	…are going too far, which should not be expected from you?	…estão a ir longe de mais, em relação a tarefas que não deveriam ser realizadas por mim?
7	…put you into an awkward position?	…me colocam numa posição incómoda?
8	…are unfair that you have to deal with them?	…é injusto ter de lidar com elas?

Note: Items 1–4 assess Unnecessary Tasks; Items 5–8 assess Unreasonable Tasks.
